# Enhanced Lutetium Ion Sorption from Aqueous Solutions Using Activated Ion Exchangers

**DOI:** 10.3390/polym16020220

**Published:** 2024-01-12

**Authors:** Talkybek Jumadilov, Khuangul Khimersen, Józef Haponiuk, Bakytgul Totkhuskyzy

**Affiliations:** 1 Bekturov Institute of Chemical Sciences, 106 Sh. Ualikhanov Str., Almaty 050010, Kazakhstan; jumadilovtalkybek@gmail.com (T.J.); huana88@mail.ru (K.K.); 2 School of Chemical Engineering, Kazakh-British Technical University, 59 Tole bi Str., Almaty 050000, Kazakhstan; 3 Institute of Natural Sciences and Geography, Abai Kazakh National Pedagogical University, 13 Dostyk Ave., Almaty 050010, Kazakhstan; 4 Department of Polymer Technology, Gdansk University of Technology, Gabriela Narutowicza 11/12, 80-233 Gdańsk, Poland; jozef.haponiuk@pg.edu.pl; 5 School of Pharmacy, Asfendiyarov Kazakh National Medical University, 94 Tole bi Str., Almaty 050012, Kazakhstan

**Keywords:** ion exchangers, interpolymer system, mutual activation, lutetium ions, sorption

## Abstract

The growing demand for rare earth elements (REE) requires the search for economically viable materials to efficiently recover REE from various solutions. Our research aims to investigate the potential of using a combination of the ion exchangers Lewatit CNP LF (in H^+^ form) and AV-17-8 (in OH^−^ form) as an interpolymer system, “Lewatit CNP LF@AV-17-8” (X:Y), with varying mass ratios of X:Y to enhance the sorption efficiency of lutetium ions from nitrate solution. During the study, we used a range of analytical methodologies, including gravimetry, ultraviolet-visible (UV-VIS) spectroscopy, and inductively coupled plasma optical emission spectroscopy (ICP-OES). Our findings demonstrate that the interpolymer system “Lewatit CNP LF@AV-17-8” (X:Y), with a mass ratio of 4:2, exhibited a significantly enhanced sorption rate of Lu^3+^ ions (42%) compared to the individual Lewatit CNP LF (6:0) (25%) and the individual AV-17-8 (0:6) (21%) over a 48 h period. Moreover, this interpolymer system has demonstrated notable conformity to the Freundlich adsorption model, highlighting its performance as an effective sorbent for lutetium (III) ions. Notably, our study presents a novel utilization of the interpolymer system “Lewatit CNP LF@AV-17-8” (4:2), with an adsorption capacity of 221.05 mg/g, to enhance the recovery of lutetium ions. The research findings demonstrate its potential for enhancing the recovery of REE.

## 1. Introduction

In the dynamic field of modern technology, certain elements have garnered increased attention due to their unique features and diverse applications. The rare earth elements (REE), comprising 15 lanthanides (including lutetium), form a group of 17 components (with yttrium and scandium) found on the periodic table. These elements play a crucial role in both the scientific and industrial sectors [1,2]. Lutetium, among other elements, has found extensive use in various modern technologies, ranging from smartphones and hybrid vehicles to advanced medical devices in nuclear medicine and clean energy solutions [3,4,5]. Lutetium also possesses the ability to capture thermal neutrons, enabling its transformation into an isotope with exceptional therapeutic potential for radioisotope-based cancer treatment technologies [6,7,8,9].

Despite its valuable properties, lutetium presents challenges in terms of production when obtained from ores [10,11,12]. However, an alternative approach to acquiring this REE involves utilizing industrial solutions that often contain valuable components. Hydrometallurgical methods show promise in the production of REEs, as they offer controlled recovery rates and the ability to produce high-purity products [13,14,15]. Therefore, the development of effective sorption technologies for lutetium ion recovery from various solutions is essential for promoting the sustainable utilization of natural resources and advancing processing techniques.

By implementing efficient sorption technologies, the recovery of REE can be carried out more effectively, leading to a reduction in reliance on traditional ore extraction methods and contributing to sustainability efforts. This transition towards advanced recovery techniques aligns with the broader objective of optimizing resource utilization and minimizing environmental impact, thereby ensuring a more sustainable future for lutetium-dependent industries. Currently, the urgent challenge in hydrometallurgy is the development of effective sorption technologies for REE recovery from wastewater solutions [16,17,18,19,20]. Various adsorbents, such as clays [21,22,23], functionalized magnetic chitosan nanoparticles [24,25,26], carbon adsorbents [27,28,29,30], biosorbents [31,32,33], hydrogels [34,35,36,37,38], and ion exchangers [39,40,41,42], can be used to recover various metal ions from aqueous solutions. The recovered metals have wide applications in catalysis, pyrotechnics, coal chemistry, and other fields [43,44,45,46,47,48]. However, the practical application of recently developed polymeric sorbents has been limited [49,50,51]. This is primarily due to the limited suitability of most ion exchangers to be used in complex solutions. 

Ion exchange materials play a crucial role in the sorption of REEs due to their unique and highly selective properties. The recovery of REEs from industrial or primary sources holds significant economic and environmental value in various sectors such as mining, electronics, and renewable energy. In contrast, traditional methods of REE extraction, such as solvent extraction and precipitation, often result in substantial waste generation and environmental concerns. On the other hand, ion exchange processes are generally more environmentally friendly, producing fewer waste byproducts and reducing pollution. Additionally, these processes can be easily scaled up for industrial applications, making them suitable for large-scale REE extraction and recovery efforts. Given the increasing demand for REEs in modern technologies, the development of efficient recovery methods is essential for conserving the finite lutetium resources [52,53,54]. The main aim of this study is to take a substantial step forward in the advancement of an effective and groundbreaking approach for the recovery of lutetium ions from aqueous solutions. The novelty of our study lies in the first-time utilization of an interpolymer system, specifically “Lewatit CNP LF@AV-17-8” (X:Y), for the sorption of lutetium ions from an aqueous medium. This specific application has not been previously studied.

## 2. Materials and Methods

### 2.1. Materials and Equipment

The materials used in this study were weakly acidic cation exchanger Lewatit CNP LF (in H^+^ form) and strongly basic anion exchanger AV-17-8 (in OH^−^ form). Lewatit CNP LF (LANXESS Deutschland GmbH, Cologne, Germany) is a macroporous cross-linked polyacrylate-based cation exchanger with a granule size of 0.315–1.600 mm. AV-17-8 (Azot, Cherkasy, Ukraine) is a styrene and divinylbenzene copolymer with a granule size of 0.315–1.250 mm.

The reagent used in the study, lutetium (III) nitrate hydrate (Sigma-Aldrich, Saint Louis, MO, USA), was used to prepare a lutetium-containing solution with a concentration of 100 mg/L. The Arsenazo III metal indicator (Merck KGaA, Darmstadt, Germany) reagent, a color-forming reagent to prepare detectable forms of the lutetium ion complexes in the samples, was also used. Perchloric acid (HClO_4_, ACS reagent, 70%) (Sigma-Aldrich, Darmstadt, Germany) was used to prepare the standard solution, and nitric acid (HNO_3_, ACS reagent, 70%) (Sigma-Aldrich, Saint Louis, MO, USA) was used to prepare 2 M nitric acid solution as the eluent for the desorption of lutetium ions.

The masses of the materials were determined using an analytical balance, Shimadzu AY220 (Shimadzu Corporation, Kyoto, Japan). The optical densities of the solutions were measured using a Jenway-6305 UV-VIS spectrophotometer (Cole-Parmer, Jenway, York, UK) to find the Lu^3+^ concentrations in aliquoted samples. The presence of residual lutetium ions in the aliquoted samples was detected using the Optima 8300DV Duo inductively coupled plasma-optical emission spectrometer (PerkinElmer, Waltham, MA, USA). The measurement errors were found to be less than 1%.

### 2.2. Preparation of the Interpolymer System “Lewatit CNP LF@AV-17-8” (X:Y)

In order to develop an effective interpolymer system that can interact remotely in aqueous media, it is crucial to carefully select polymers with compatible chemical properties. This is necessary to ensure that the polymers can interact despite their different (acidic or basic) natures [55,56]. Our previous research [57,58] has demonstrated that an interpolymer system consisting of two cross-linked polymers with active functional groups can be effective. These polymers are placed in a common solution, but without direct contact, as an interpolymer system, Lewatit CNP LF@AV-17-8” (X:Y). The aforementioned ion exchangers were accurately weighed (Table 1) and placed into polypropylene bags (50 mm × 100 mm) according to their X:Y mass ratios. The interpolymer system “Lewatit CNP LF@AV-17-8”, with mass ratios of X:Y equal to 6:0, 5:1, 4:2, 3:3, 2:4, 1:5, and 0:6, was prepared for further investigation.

### 2.3. Activation of the Interpolymer System “Lewatit CNP LF@AV-17-8” (X:Y) 

In an aqueous environment, ion exchangers possess the capability to release or accept protons in the form of oxonium ions (H_3_O^+^), depending on the structural composition of the polymers and the pH of the solution (which relates to the acid–base properties of the solution). Typically, these polymers contain functional groups that are inherently either acidic or basic, and their dissociation process adheres to an acid–base equilibrium.

When placed into aqueous media, ion exchangers undergo a sequential dissociation process. Initially, the functional group of the ion exchanger attracts the ion, initiating the first step. Subsequently, the second step occurs, during which the targeted ion is exchanged with a counterion that was previously bound to the ion exchanger. As a result, the released counterion is discharged into the surrounding solution [59].

**Figure 1:**
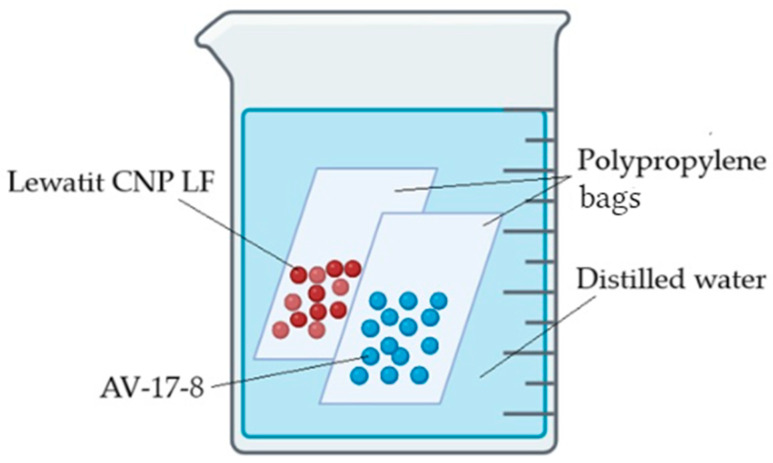
Representation of the activation process of the interpolymer system “Lewatit CNP LF@AV-17-8” (X:Y) through remote interaction.

The activation of the interpolymer system is urgent in order to enhance the ionization state of the ion exchangers. This is achieved by modifying their conformational and electrochemical properties through a process of remote interaction. To accomplish this, polypropylene bags containing ion exchangers are placed within a glass container filled with distilled water. Then, the polypropylene bags are positioned approximately 1–2 cm apart, forming the interpolymer system “Lewatit CNP LF@AV-17-8” (X:Y) with a mass ratio of X:Y (Figure 1).

Polypropylene bags are commonly used in biomedical applications due to their exceptional qualities (chemical resistance, mechanical strength, and biocompatibility). Their compatibility with aqueous media is a crucial factor in their performance. Polypropylene, being a non-polar thermoplastic polymer, exhibits hydrophobic properties, making it resistant to water. As a result, when polypropylene bags are exposed to an aqueous environment, they maintain chemical and physical stability due to their low solubility and exceptional resistance to water [60].

**Scheme 1:**
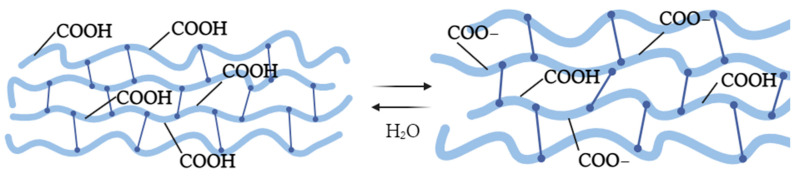
The dissociation of the ion exchanger Lewatit CNP LF.

**Scheme 2:**
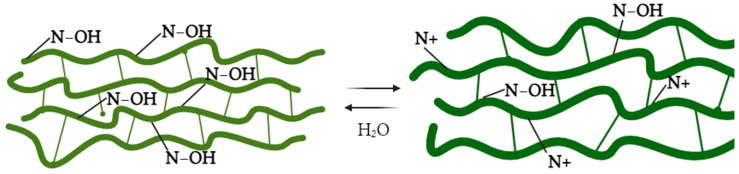
The dissociation of the ion exchanger AV-17-8.

There are two main dissociation steps of the ion exchangers Lewatit CNP LF and AV-17-8 that occur in the aqueous medium:

1. The cation exchanger Lewatit CNP LF (in H^+^ form) dissociates in an aqueous solution according to the Scheme 1:

2. The anion exchanger AV-17-8 (in OH^−^ form) dissociates in an aqueous solution according to the Scheme 2:

### 2.4. Determination of the Polymer Chain Binding Properties

The polymer chain binding rate (*θ*) specifies the number of units around the lutetium ion. It directly depends on the ionization rate of the ion exchangers Lewatit CNP LF and AV-17-8 during their mutual interaction in the interpolymer system “Lewatit CNP LF@AV-17-8” (X:Y). The value *θ* was calculated according to Equation (1) [61,62]:

(1) $$\theta =\frac{\vartheta_{sorbed}}{\vartheta_{is}}\times 100\%$$

where *ϑ_sorbed_* is the amount of sorbed lutetium ions (in mol), and *ϑ_is_* is the amount (in mol) of the interpolymer system “Lewatit CNP LF@AV-17-8” (X:Y).

### 2.5. Plotting a Calibration Curve

**Figure 2:**
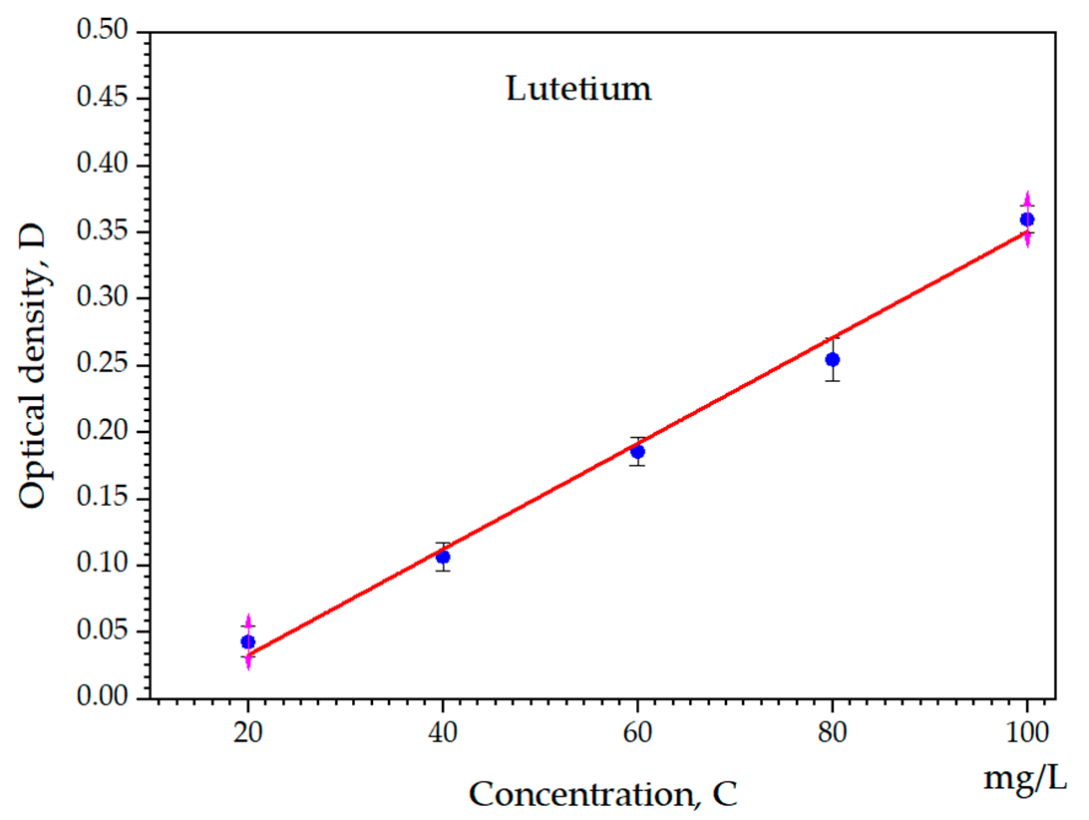
The calibration curve to determine the concentrations of lutetium (III) in the tested solutions (r^2^ value 0.99354).

The method for determining the lutetium ions was based on the formation of a colored complex compound using the organic analytical reagent arsenazo III with the lutetium ions. To obtain an analytical form, it was necessary to introduce a colored reagent such as arsenazo III, which is a bisazo-derivative of chromotropic acid [34,63]. The calibration curve for determining the concentrations of lutetium (III) ions in the tested solutions is presented in Figure 2. The calibration curve was plotted using the “Origin 2018” software.

### 2.6. Determination of the Sorption Rate of the Lutetium Ions by the Interpolymer System “Lewatit CNP LF@AV-17-8” (X:Y)

In the experiments, lutetium (III) nitrate solution with a concentration of 100 mg/L was prepared and distributed into seven laboratory beakers, each with a volume of 100 mL. The ion exchangers Lewatit CNP LF and AV-17-8 were placed separately within the polypropylene mesh in a shared beaker, along with the solution, following specific mass ratios (6:0, 5:1, 4:2, 3:3, 2:4, 1:5, and 0:6) to form the interpolymer system “Lewatit CNP LF@AV-17-8” (X:Y). Spectrophotometric analysis was conducted by extracting a 1 mL aliquot from each solution at designated time intervals (1, 2, 4, 6, 12, 24, 36, and 48 h), resulting in a total of 56 aliquots in the final step. Then, each aliquot (1 mL) with an unknown concentration of the analyte was transferred into a 50 mL volumetric flask. Subsequently, 12 mL of a 0.015% arsenazo solution and 2 mL of a 0.08 M perchloric acid solution were added to each flask. The volume of each solution was then adjusted to 50 mL with distilled water. Measurements were initiated after 15 min. The reference solution contained all the previously mentioned constituents, with the exception of the analyte.

The unknown concentrations were determined for each signal by obtaining the value of the analytical signal (D) and using a standard solution of lutetium. The optical densities (D) of solutions containing lutetium ions were measured using the Jenway-6305 spectrophotometer across a wavelength range of 198 to 1000 nm. Each measurement was conducted three times, and the average value was calculated. The sorption rate (η) was calculated using Equation (2), as follows:

(2) $$\eta =\frac{C_{initial}-C_{residual}}{C_{initial}}\times 100\%$$

where *C_initial_* and *C_residual_* are the initial and residual concentrations (in g/L) of lutetium ions in the solutions.

### 2.7. Determination of the Desorption Rate of the Lutetium Ions from the Interpolymer System “Lewatit CNP LF@AV-17-8” (4:2)

The study focused on investigating the desorption kinetics in the interpolymer system “Lewatit CNP LF@AV-17-8” (4:2), which was found to be the most efficient system for the sorption of lutetium ions among the mass ratios examined. The procedure involved the extraction of lutetium ions that were adsorbed on the surface of the interpolymer system “Lewatit CNP LF@AV-17-8” (4:2) using 2 M nitric acid as the eluent. Nitric acid was chosen as the eluent due to its strong affinity for the adsorbed rare earth metals. The desorption rate (*R*) was determined using Equation (3).

(3) $$R=\frac{m_{desorbed}}{m_{sorbed}}\times 100\%$$

where $m_{desorbed}$ and $m_{sorbed}$ are the desorbed and sorbed masses of lutetium (in mg).

### 2.8. Inductively Coupled Plasma Optical Emission Spectroscopy (ICP-OES) Analysis

ICP-OES analysis was utilized as a highly advanced analytical technique to accurately detect and measure the presence of lutetium ions in the aliquoted samples. This method involves heating the sample to create plasma, which is then stimulated by radiofrequency radiation. The resulting emission of light is analyzed by a spectrometer, with each spectral line corresponding to a specific element in the sample. By measuring the intensity of these spectral lines, we were able to determine the concentration of lutetium ions in the aliquoted samples. The elemental composition of the sample was assessed by evaluating the intensities of these spectral lines. For a visual representation of our findings, please refer to the Results and Discussion section, which includes graphical representations.

### 2.9. Freundlich Adsorption Isotherm Model

The adsorption isotherms were determined using the Freundlich model, which is well-suited for heterogeneous surfaces. According to this model, an increase in the concentration of metal particles in the liquid phase leads to a higher concentration of ionic particles being adsorbed onto the surface of the solid substance (polymer) [64]. The model also suggests that the adsorption energy decreases exponentially as the adsorption centers of the adsorbent reach their maximum capacity [65]. This relationship can be described by the following linearized Equation (4):

(4) $${logQ}_{e}={logK}_{f}+\frac{1}{n}{logC}_{e}$$

where *K_f_* is the constant that relates to the sorption capacity of the adsorbent (the interpolymer system) for the species, and *n* is the constant that relates to the sorption intensity (the effect of lutetium concentration). Adsorption conditions are favorable when *n* > 1 [66]. The plots of *logQ_e_* vs. *logC_e_* exhibit a linear relationship with a slope of 1/*n* and an intercept of *logKf*.

## 3. Results and Discussion

### 3.1. Impact of the Polymers Activation Mechanism in the Interpolymer System “Lewatit CNP LF@AV-17-8” (X:Y) on the Sorption of the Lutetium Ions

The mechanism of remote interaction involves the exclusion of direct interaction between the acidic polymer (Lewatit CNP LF) and the basic polymer (AV-17-8) due to the dynamic surface morphology. To explain this phenomenon, we can examine the dissociation of the ion exchangers Lewatit CNP LF (Scheme 1) and AV-17-8 (Scheme 2), which leads to the generation of free H_3_O^+^ and OH^−^ ions in the shared solution, respectively. These ions, in turn, create weakly dissociated water molecules that activate and stabilize the functional groups of polyelectrolytes through intramolecular interactions. The Pearson’s Hard and Soft Acid and Base (HSAB) theory [67] also aligns with this phenomenon. According to this theory, hard acid H^+^ and hard base OH^−^ form a water molecule, which activates and stabilizes ion exchanger groups [68]. Consequently, the concentrations of H_3_O^+^ and OH^−^ ions are significantly higher around Lewatit CNP LF and AV-17-8, respectively. This gradient potentially reduces the concentration of neutral water around the ion exchangers in the interpolymer system, compared to when they are used individually. As a result, it enhances the dissociation of counter ions from the ionic groups of the polyelectrolyte. Moreover, according to Le Chatelier’s principle [69], it is possible for both polymers to undergo additional dissociation. As a consequence of this process, the Lewatit CNP LF and AV-17-8 macromolecules experience unfolding of their links and destruction of the intramolecular bonds, primarily due to the electrostatic repulsion of the -COO^−^ and N^+^ groups. When the intrachain links are disrupted, both macromolecules undergo additional unfolding. The outcome of the remote interaction is the mutual activation of the initial ion exchangers in the interpolymer system, resulting in their transition to a highly ionized state. This, in turn, significantly enhances the sorption properties of polyacid Lewatit CNP LF and polybase AV-17-8 in relation to lutetium ions in the interpolymer system “Lewatit CNP LF@AV-17-8” (X:Y).

The highest sorption of lutetium ions by the interpolymer system “Lewatit CNP LF@AV-17-8” (4:2) may involve complex interactions influenced by the electronic structure of Lu, particularly its filled 4f orbital and partially filled 5d orbital. These interactions are crucial for understanding the sorption mechanism and can provide insights into how similar processes might occur with other lanthanides or in different chemical environments [70].

### 3.2. Sorption Characteristics of the Interpolymer System “Lewatit CNP LF@AV-17-8” (X:Y) in Relation to Lutetium Ions

**Figure 3:**
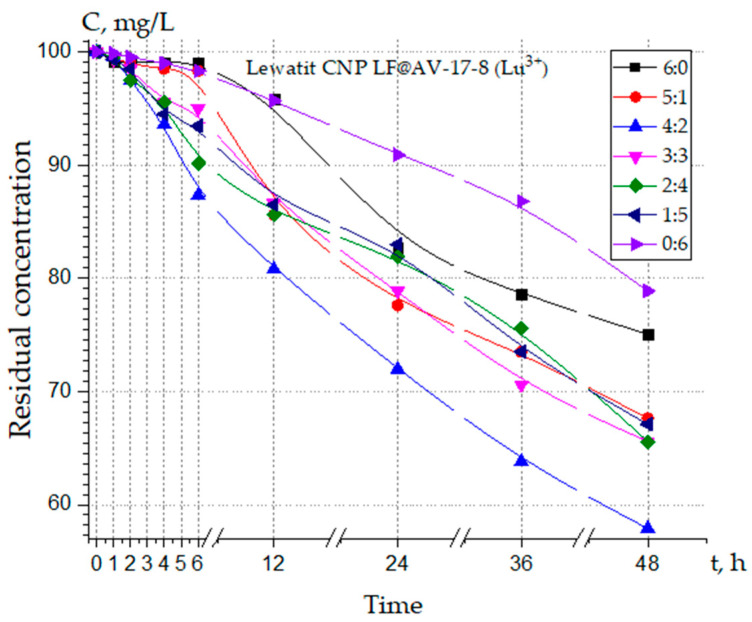
The dynamic of the sorption of lutetium ions by the interpolymer system “Lewatit CNP LF@AV-17-8” (X:Y) as a function of time. Conditions: temperature of 25 °C.

The sorption dynamics of lutetium ions by the interpolymer system “Lewatit CNP LF@AV-17-8” (X:Y) are illustrated in Figure 3. 

The observed changes in Lu^3+^ concentration indicate the complex interaction between ion-exchange kinetics and chemical interactions. These interactions occur when lutetium ions interact with the interpolymer system. As shown in Figure 3, after 48 h of interaction, certain ratios of the ion exchangers in the interpolymer system “Lewatit CNP LF@AV-17-8” (X:Y) (i.e., 5:1, 3:3, 1:5, and 0:6) exhibited slightly higher sorption activity compared to the individual Lewatit CNP LF cation exchanger (6:0). Moreover, the highest rate of lutetium ion sorption was observed at a ratio of 4:2 within 48 h of the interaction. This might be because, at this ratio (4:2), both the Lewatit CNP LF and AV-17-8 ion exchangers in their interpolymer system became highly ionized due to their mutual activation, making them maximally complementary to the lutetium ions.

### 3.3. Determination of the Polymer Chain Binding Properties

As previously mentioned, the polymer chain binding rate (*θ*, %) specifies the number of units around the lutetium ion and is directly dependent on the ionization rate of the Lewatit CNP LF and AV-17-8 ion exchangers in the interpolymer system “Lewatit CNP LF:AV-17-8” (X:Y) during remote interaction in the aqueous medium.

Table 2 illustrates the binding rate (*θ*) of the polymer chain in the interpolymer system “Lewatit CNP LF@AV-17-8” (X:Y) in relation to lutetium ions. The highest binding of lutetium ions by this interpolymer system was observed within 48 h of the interaction. The interpolymer system “Lewatit CNP LF@AV-17-8” (X:Y) showed increased polymer chain binding rates at specific X:Y ratios, namely, 4:2 (3.91%), 3:3 (3.26%), and 2:4 (3.32%). These findings suggest a significant level of macromolecule ionization, indicating the synergistic activation of the Lewatit CNP LF and AV-17-8 ion exchangers in the aqueous environment.

### 3.4. Determination of the Sorption Rate of Lutetium Ions

**Figure 4:**
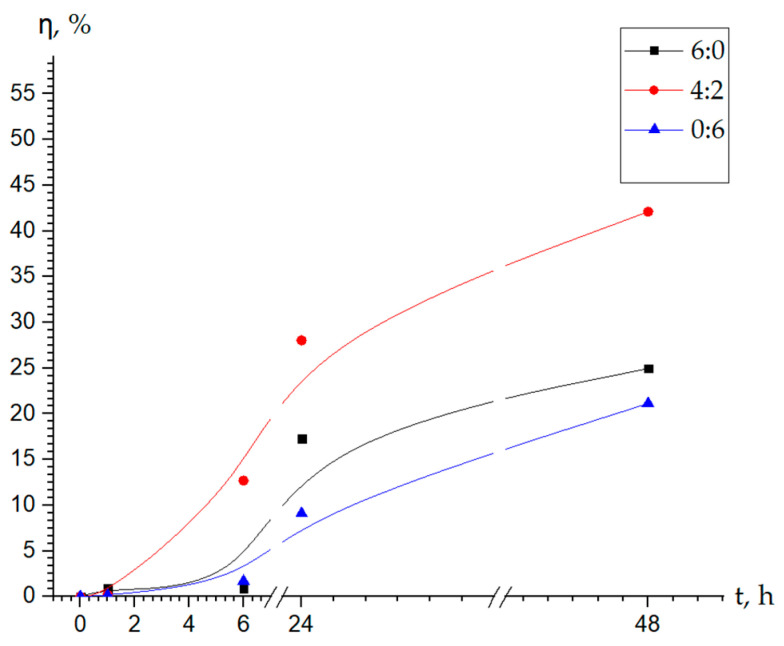
The sorption rate of lutetium ions by the interpolymer system “Lewatit CNP LF@AV-17-8” (4:2), as compared with the individual Lewatit CNP LF (6:0) and AV-17-8 (0:6) ion exchangers.

Figure 4 illustrates that the interpolymer system “Lewatit CNP LF@AV-17-8” (4:2) exhibited the highest sorption activity towards lutetium ions. Consequently, this specific interpolymer system was selected for further comprehensive comparison with the initial Lewatit CNP LF (6:0) and initial AV-17-8 (0:6) ion exchangers based on the data in Figure 3. In Figure 4, it can be observed that, after 48 h of the interaction, the “Lewatit CNP LF@AV-17-8” (4:2) interpolymer system exhibited the highest rate of lutetium ion sorption, at 42%, after the same interaction duration. In comparison, the initial Lewatit CNP LF (6:0) and initial AV-17-8 (0:6) ion exchangers showed percentages of 25% and 21%, respectively.

The Lewatit CNP LF and AV-17-8 ion exchangers, which are part of the “Lewatit CNP LF:AV-17-8” interpolymer system with a molar ratio of 4:2, adopted the optimal conformation for the maximum sorption of lutetium ions. The remote interaction between Lewatit CNP LF and AV-17-8 in an aqueous solution activated the ion exchangers in the interpolymer system, leading to a subsequent transition to a highly ionized state. This transition resulted in a significant increase in the sorption of lutetium ions.

Table 3 demonstrates the influence of solution pH on the maximum sorption capacity of various sorbents for lutetium ions. According to the findings presented in Table 2, some studies have indicated that the optimal pH range for achieving maximum lutetium sorption lies between 4.0 and 5.0. This suggests that the sorption characteristics of different sorbents may be directly influenced by the ionic form of lutetium in aqueous solutions. In our study, we observed that the interpolymer system “Lewatit CNP LF@AV-17-8” (4:2) exhibited maximum sorption at pH 4.7. This pH influences the surface charge of the interpolymer system “Lewatit CNP LF@AV-17-8” (4:2). At pH 4.7, the surface groups on the sorbent material may become more ionized, enhancing their ability to bind with the target lutetium ions. Our polymer sorbent exhibited a maximum sorption capacity of 221.05 mg/g for lutetium ions.

### 3.5. Kinetics of Lutetium Desorption from the Interpolymer System “Lewatit CNP LF@AV-17-8” (4:2)

**Figure 5:**
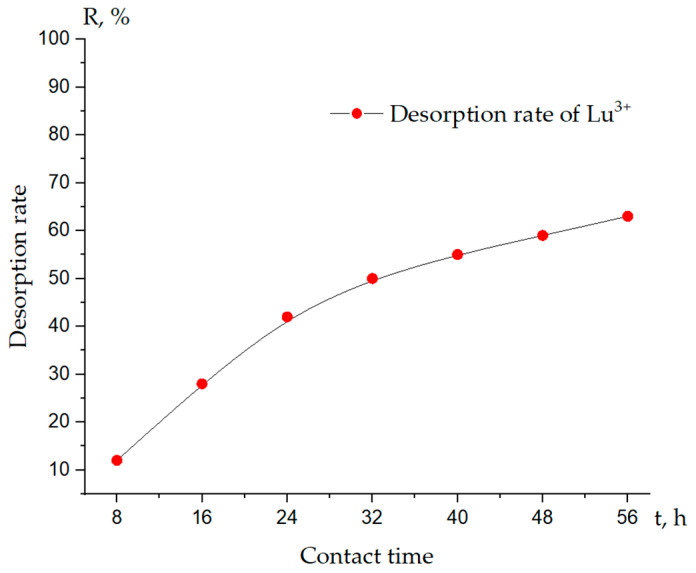
Kinetics of lutetium desorption from the interpolymer system “Lewatit CNP LF@AV-17-8” (4:2). Conditions: temperature of 25 °C.

To investigate the desorption of lutetium ions from the interpolymer system “Lewatit CNP LF@AV-17-8” (4:2), a 2 M nitric acid solution was prepared and used as the desorbing agent (eluent). The desorption kinetics of the lutetium ions are illustrated in Figure 5. The desorption rate of lutetium ions (R (Lu) %) was determined using Equation (3). Figure 5 demonstrates the desorption process, which resulted in the highest value of lutetium ion desorption (63%) after a duration of 56 h.

### 3.6. ICP-OES Analysis of the Residual Concentration of Lutetium Ions

Inductively coupled plasma optical emission spectroscopy (ICP-OES), as a powerful analytical technique, was used in our research to quantify the presence of lutetium ions in the selected samples. In our study, ICP-OES was employed as an additional analytical technique to detect the residual concentration of lutetium ions (after sorption) in aliquoted samples.

**Figure 6:**
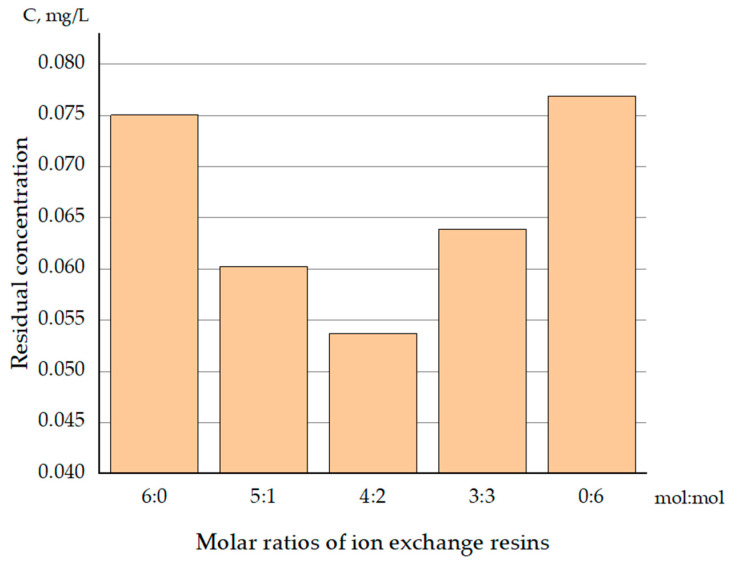
ICP-OES analysis of the residual concentration of lutetium ions in tested aliquots after 48 h of sorption.

The data obtained in this study underwent further validation through ICP-OES analysis. Figure 6 presents the results of the ICP-OES analysis for different ratios of “Lewatit CNP LF@AV-17-8” (X:Y). Specifically, the ratios tested were 6:0, 5:1, 4:2, 3:3, and 0:6. Notably, the interpolymer system “Lewatit CNP LF@AV-17-8” (4:2) resulted in the lowest residual lutetium ion concentration, indicating the highest sorption efficiency in the tested aliquoted samples. The experimental results confirm the advanced sorption capabilities of the interpolymer system “Lewatit CNP LF@AV-17-8” (4:2) for lutetium (III) ions from the simulated wastewater solution.

### 3.7. Freundlich Adsorption Model for the Interpolymer System “Lewatit CNP LF@AV-17-8” (4:2)

The understanding and control of sorption phenomena are crucial in the rare earth element (REE) recovery and separation processes. The ability of sorbents to effectively adsorb specific ions from solutions is often characterized by adsorption isotherms. Among these, the Freundlich adsorption isotherm stands out as a versatile model that describes non-ideal multilayer adsorption processes.

**Figure 7:**
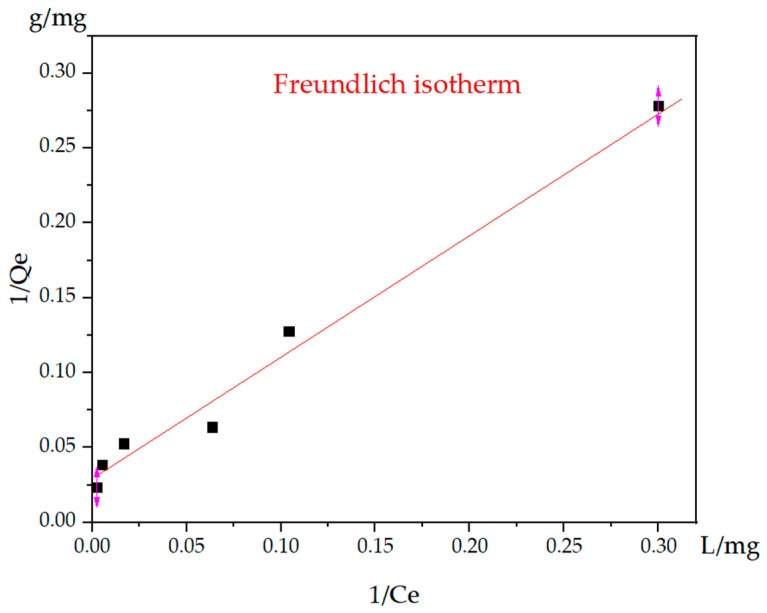
Freundlich adsorption isotherm of the interpolymer system “Lewatit CNP LF@AV-17-8” (4:2) (Lu^3+^).

In this study, we investigate the use of the Freundlich model to evaluate the sorption characteristics of the interpolymer system “Lewatit CNP LF@AV-17-8” (4:2) for lutetium (III) ions (Figure 7). This system has demonstrated a notable ability to adsorb REEs from solutions, and their adherence to the Freundlich line signifies their effectiveness in these processes. Our research illuminates the vast potential of this interpolymer system in the field of rare earth element recovery and its wider applications in ion exchange and separation processes.

The analysis of Freundlich adsorption isotherm (Figure 7) conducted on the interpolymer system “Lewatit CNP LF@AV-17-8” (4:2) for lutetium (III) ions has provided valuable insights into their sorption behavior. Notably, this system has shown a remarkable adherence to the Freundlich adsorption model. The fact that our interpolymer systems closely follow the linear relationship outlined by this model underscores their effectiveness as sorbents for lutetium (III) ion recovery. This adherence to the Freundlich model suggests that the interpolymer system can offer versatile and efficient sorption capabilities across various compositions.

## 4. Conclusions

In this study, we have thoroughly examined the intricate dynamics of lutetium ion sorption within the interpolymer system “Lewatit CNP LF@AV-17-8” (X:Y). Our research has yielded significant insights into the influences of various factors on the sorption process. This sheds light on the remarkable potential of the interpolymer system for REE recovery and separation.

The concept of remote interaction, driven by the dynamic surface morphology of acidic and basic polymers, has been elucidated. This interaction yielded H_3_O^+^ and OH^−^ ions, which activate and stabilize functional groups, thereby enhancing ion exchange capabilities. The Pearson’s Hard and Soft Acid and Base (HSAB) theory and Le Chatelier’s principle align with this phenomenon, further corroborating the mutual activation of ion exchangers within the system.

Our findings highlight the dependence of Lu^3+^ ion sorption on the ratio of Lewatit CNP LF to AV-17-8. A specific ratio, such as 4:2, demonstrates significantly higher sorption activity toward lutetium compared to individual (0:6 and 6:0) ion exchangers. This underscores the critical role of polymer composition in optimizing sorption efficiency. The adherence of the interpolymer system “Lewatit CNP LF@AV-17-8” (4:2) to the Freundlich adsorption isotherm signifies its effectiveness for lutetium ion recovery. 

In conclusion, our research highlights the significant potential of the interpolymer system “Lewatit CNP LF@AV-17-8” (4:2), with an adsorption capacity of 221.05 mg/g, as a potentially efficient sorbent for the recovery of Lu^3+^ ions from simulated wastewater solutions. The distinctive interactions of various factors, including polymer activation, sorption characteristics, polymer chain binding rate, thermal stability, and adherence to the Freundlich adsorption model, renders this system a valuable resource in the realm of REE recovery and separation processes.

For future research, to evaluate the feasibility of extracting Lu^3+^ ions from secondary sources on an industrial scale, additional studies and developments are essential. Research should focus on selectively extracting lutetium ions from mixed REE-containing solutions using an appropriate interpolymer system in order to advance the technology. The effect of ion radii on the selective recovery and separation of lanthanides from REE-containing solutions should be studied. These research areas are crucial for enhancing the understanding and practical application of the REE sorption process.

## Figures and Tables

**Table 1:** Molar and mass ratios of the ion exchangers in the interpolymer system “Lewatit CNP LF@AV-17-8” (X:Y).

Molar Ratio of Lewatit CNP LF@AV-17-8 (X:Y)	Mass Ratio of Lewatit CNP LF@AV-17-8 (X:Y) ^a^
6:0	0.200 g:0.000 g
5:1	0.170 g:0.025 g
4:2	0.140 g:0.050 g
3:3	0.100 g:0.075 g
2:4	0.070 g:0.100 g
1:5	0.035 g:0.125 g
0:6	0.000 g:0.150 g

^a^ The ratio of the components was determined using the gravimetric method.

**Table 2:** The rate of polymer chain binding (*θ*, %) of lutetium cations by the interpolymer system “Lewatit CNP LF@AV-17-8” (X:Y).

Molar Ratio of Lewatit CNP LF@AV-17-8 (X:Y)	Mass Ratio of Lewatit CNP LF@AV-17-8 (X:Y) ^a^	θ after 1 h	θ after 6 h	θ after 24 h	θ after 48 h
6:0	0.200 g:0.000 g	0.08%	0.08%	1.54%	2.23%
5:1	0.170 g:0.025 g	0.41%	0.54%	2.04%	2.95%
4:2	0.140 g:0.050 g	0.15%	1.18%	2.60%	3.91%
3:3	0.100 g:0.075 g	0.06%	0.47%	2.00%	3.26%
2:4	0.070 g:0.100 g	0.16%	0.95%	1.74%	3.32%
1:5	0.035 g:0.125 g	0.06%	0.64%	1.68%	3.24%
0:6	0.000 g:0.150 g	0.01%	0.17%	0.91%	2.12%

Conditions: temperature of 25 °C. ^a^ Ratio of the components determined by the gravimetry method.

**Table 3:** The impact of solution pH on the maximum sorption capacity of different sorbents for lutetium ions.

Sorbents	pH Range	Optimum pH	Maximum Sorption Capacity (mg/g)	References
Transcarpathian clinoptilolite	4.0–13.0	10.0	9.37	[71]
Mesoporous MCM-41 silica impregnated with Cyanex 272	1.0–3.0	2.5	44.00	[72]
Alumina–silica-based composite	1.0–4.0	4.0	129.77	[73]
Organic ligand-based composite hybrid material	4.0–5.0	4.0	171.76	[74]
Interpolymer system “Lewatit CNP LF@AV-17-8” (4:2)	4.0–5.0	4.7	221.05	Current study

## Data Availability

The data presented in this study are available upon request from the corresponding author.
